# The Association of Aging and Aerobic Fitness With Memory

**DOI:** 10.3389/fnagi.2018.00063

**Published:** 2018-03-09

**Authors:** Alexis M. Bullock, Allison L. Mizzi, Ana Kovacevic, Jennifer J. Heisz

**Affiliations:** Department of Kinesiology, McMaster University, Hamilton, ON, Canada

**Keywords:** high-interference memory, general recognition memory, lifespan, cognitive decline, aerobic fitness

## Abstract

The present study examined the differential effects of aging and fitness on memory. Ninety-five young adults (YA) and 81 older adults (OA) performed the Mnemonic Similarity Task (MST) to assess high-interference memory and general recognition memory. Age-related differences in high-interference memory were observed across the lifespan, with performance progressively worsening from young to old. In contrast, age-related differences in general recognition memory were not observed until after 60 years of age. Furthermore, OA with higher aerobic fitness had better high-interference memory, suggesting that exercise may be an important lifestyle factor influencing this aspect of memory. Overall, these findings suggest different trajectories of decline for high-interference and general recognition memory, with a selective role for physical activity in promoting high-interference memory.

## Introduction

Aging is associated with progressive changes in brain structure that gradually impair essential cognitive functions, including memory (Park and Reuter-Lorenz, 2009; Lockhart and DeCarli, 2014; Madan and Kensinger, 2017). Numerous mechanisms have been proposed to underlie memory decline, such as altered plasticity, connectivity and excitability (Ash and Rapp, 2014; Leal and Yassa, 2015). One change of particular interest is the reduction of hippocampal neurogenesis (Kuhn et al., 1996; McDonald and Wojtowicz, 2005; Montaron et al., 2006; Spalding et al., 2013) and the observation that age-associated declines in neurogenesis and memory can be partly rescued in animals that engage in aerobic exercise (van Praag et al., 2005). Aerobic exercise also increases hippocampal blood volume in humans (Pereira et al., 2007; Maass et al., 2016). Critically, this suggests that physical activity in older humans may be associated with better memory performance. However, some aspects of memory processing may be more associated with aerobic fitness than others. The present study compared age-related differences for two critical memory processes: high-interference memory and general recognition memory.

High-interference memory represents the ability to discriminate between highly similar yet distinct items (Yassa and Stark, 2011; Heisz et al., 2017). Computational models suggest newborn cells within the dentate gyrus of the hippocampus are recruited to encode novel stimuli and reduce interference between representations of highly similar stimuli (Finnegan and Becker, 2015; Becker, 2017). The underlying neural mechanism for this process is termed pattern separation, which allows for successful performance on tasks with a high-interference memory component (Becker, 2017). In animal models, high-interference memory can be improved by stimulating neurogenesis (Sahay et al., 2011) and is impeded by interfering with neurogenesis (Clelland et al., 2009; Niibori et al., 2012). Younger adults (YA) have better high-interference memory than older adults (OA; Stark et al., 2013), therefore, the decline in hippocampal neurogenesis that occurs with aging may impact high-interference memory (Kuhn et al., 1996). However, the importance of neurogenesis in preserving high-interference memory with age is not fully understood and the relationship between neurogenesis and improved memory has not been consistently demonstrated (Bizon and Gallagher, 2003; Filipkowski and Kaczmarek, 2018). A surprising finding from one study was that deficits in high-interference memory appeared to plateau at ~60 years of age in some individuals but continued to decline in others (Stark et al., 2013). Critically, this does not fit with evidence that the hippocampal structure gradually atrophies years beyond that age (Ziegler et al., 2012; Leal and Yassa, 2015), and suggests that there may be important individual differences in age-related decline of high-interference memory, which have yet to be identified.

General recognition is another aspect of memory that represents the ability to discriminate novel stimuli from those previously encountered (Mandler, 1980). Unlike high-interference memory, general recognition memory may not be as dependent on hippocampal neurogenesis (Yonelinas et al., 2005) because it also recruits frontal and parietal regions for processing (Neufang et al., 2006). The recruitment of a more distributed network for processing by general recognition may mean that this aspect of memory is less affected by the age-related decline (Mitchell and Bruss, 2003; Danckert and Craik, 2013; Holden et al., 2013; Stark et al., 2013). Indeed, YA and OA have been shown to have similar performance on general recognition memory tasks (Stark et al., 2013).

Individual differences influencing age-related decline of memory extend beyond biological aging to include lifestyle factors, such as physical activity (Barnes et al., 2003; Fenesi et al., 2017; Martin Ginis et al., 2017). Indeed, in animal models, aerobic exercise improves high-interference memory (Creer et al., 2010). In YA, high-intensity exercise improves aerobic fitness and high-interference memory but not general recognition memory (Déry et al., 2013; Heisz et al., 2017; Suwabe et al., 2017a,b). However, these associations have yet to be examined in OA and importantly, the results may differ by age. Specifically, compared to younger animals, aerobic exercise in older animals results in wider-spread enhancements to the hippocampal structure, including increased synaptogenesis and less hippocampal degeneration (Siette et al., 2013). It follows that aerobic fitness in OA may have a broader impact on memory function; however, this has not been tested.

The present study investigated the effects of aging on high-interference memory and general recognition memory. An adapted version of the Mnemonic Similarity Task (MST) was used to assess both memory processes (Toner et al., 2009; Yassa and Stark, 2011; Stark et al., 2013; Heisz et al., 2017). We hypothesized that YA would outperform OA on high-interference memory, but not general recognition memory. We also examined individual differences in the influence of aerobic fitness on memory, with the hypothesis that OA with higher aerobic fitness would also have better memory; however, it was unclear whether the relationship would be specific to high-interference memory or whether it would be seen for both aspects of memory.

## Materials and Methods

### Participants

The present study used archival data from our laboratory collected from YA and OA; see Heisz et al. (2017) and Kovacevic (2017), respectively. Both studies received approval from the Hamilton Integrated Research Ethics Board (HiREB). Participants provided written informed consent. These studies implemented exercise interventions to measure changes in cognitive function; baseline data from participants were compared in the current analyses. Table 1 presents demographic information. YA were 17–30 years of age (*n* = 95, age *M* (SD) = 21.1 (±3.2), 58 females) and OA were 60–88 years of age (*n* = 81, age *M* (SD) = 71.4 (±5.2), 50 females). YA from McMaster University and OA from the Hamilton community were recruited using posters and online advertisements. Exclusion criteria consisted of engaging in more than 1 h of vigorous physical activity per week. OA were required to pass an exercise stress test and have no current diagnosis of cognitive impairment, as determined by a physician. Participants provided written informed consent and received an honorarium for participation.

**Table 1:** Demographic characteristics and memory performance for young adults (YA) and older adults (OA).

	YA (*n* = 95)	OA (*n* = 81)
Age (years)	21.1 (3.2)	71.4 (5.2)
Sex (F/M)	58/37	50/32
BMI (kg/m^2^)	22.5 (4.0)	29.1 (5.5)
Aerobic fitness (mL/(kg•min))	32.5 (7.5)	23.1 (6.3)
MoCA	-	26.0 (2.5)
High-interference memory	0.4 (0.2)	0.2 (0.2)
General recognition memory	0.8 (0.1)	0.8 (0.1)

*Notes. Body mass index (BMI); Montreal Cognitive Assessment (MoCA;/30). High-interference memory reflects the ability to correctly identify lure items as “Similar” [p(“Similar”|Lure image) − p(“Similar”|Foil image)]. General recognition memory reflects the ability to correctly identify repetitions items as “Old” [p(“Old”|Repeat image) − p(“Old”|Foil image)]. With the exception of sex, all data are presented as mean (SD)*.

### Procedure

#### Overview of Procedure

The baseline experimental procedure for YA and OA included cognitive tests and an aerobic fitness assessment. OA were screened for cognitive impairment using the Montreal Cognitive Assessment (MoCA), where the maximum score is 30 and higher scores indicate better cognitive functioning (Nasreddine et al., 2005; see Table 1).

#### Memory Task

**Figure 1:**
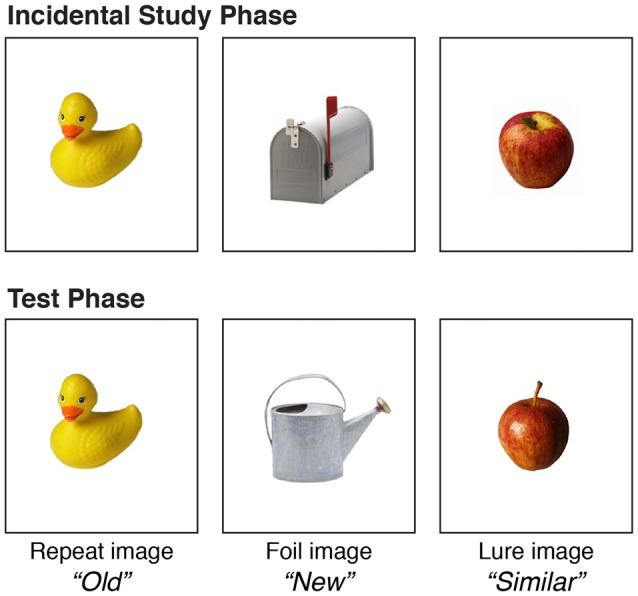
Mnemonic Similarity Task (MST). MST involves differentiating between previously learned images and novel images, some of which are highly similar, and this generates a high degree of memory interference. Following an incidental learning phase during which participants were asked to classify a sequence of 60 images of objects as indoor or outdoor, the recognition phase asked participants to judge whether each test image was an exact copy of an image already seen (30 repeat images; correct response = “Old”), highly similar but not identical to an image already seen (30 lure or decoy images; correct response = “Similar”), or completely new (30 foil images; correct response = “New”). A different stimulus set was used in the pre and post testing sessions.

An adapted version of the MST was used to assess high-interference memory and general recognition memory in YA and OA (Yassa and Stark, 2011; Stark et al., 2013; Heisz et al., 2017). See Figure 1 for a visual representation of the MST. This task involved an incidental study phase where participants were presented 60 images of everyday objects individually for 2 s on a computer screen. Participants judged each object as indoor or outdoor using the “1”and “2” key, respectively, on the number pad to aid in encoding and maintain focus. Participants then completed a test phase where they classified objects as “Old” (repetitions), “Similar” (lures), or “New” (foils) using the “1”, “2”, and “3” keys, respectively, on the number pad. “Old” objects were previously presented in the study phase, “Similar” objects were highly similar but slightly different from objects previously presented, and “New” objects were not previously presented. The test phase consisted of 90 trials including 30 repetitions, 30 lures and 30 foils.

High-interference memory was assessed as the ability to correctly identify lure items as “Similar” [p(“Similar”|Lure image) − p(“Similar”|Foil image)]. General recognition memory performance was assessed as the ability to correctly identify repetitions items as “Old” [p(“Old”|Repeat image) − p(“Old”|Foil image)]. Both high-interference memory and general recognition memory scores were corrected for response bias, as in Déry et al. (2013) and Heisz et al. (2017). Data were inspected to ensure task comprehension; participants who did not use all target keys did not understand the task and thus were removed from all memory analyses. Eight YA and one OA were excluded for this reason.

#### Aerobic Fitness

A predictive test of peak oxygen uptake (VO_2_ peak) was administered as a baseline fitness measure and the modality of exercise matched the subsequent intervention protocol. YA and OA performed different VO_2_ peak protocols to assess aerobic fitness. Prior to the VO_2_ peak test, height (m) and weight (kg) were measured and body mass index (BMI; kg/m^2^) was calculated. YA completed an incremental aerobic fitness test on a cycle ergometer using a metabolic cart (Heisz et al., 2017). OA completed a modified Bruce protocol on a motor driven treadmill with stages of 3 min in duration (Kovacevic, 2017). As designed by Willemsen et al. (2010), the Bruce protocol modifications introduced an adaptation phase of 6 min at the beginning of the test. The remainder of the assessment followed the standard Bruce protocol (Sheffield and Roitman, 1976; Willemsen et al., 2010). VO_2_ peak in OA was estimated using the following formula, with a weighting factor of 1 for men and 2 for women (Bruce et al., 1973): VO_2_ peak = 6.70 − 2.82 (weighting factor for sex) + 0.056 (duration in seconds). The 6-min introduction phase from the Bruce protocol modifications was not included in this calculation. Eleven OA completed the Single Stage Treadmill Walking Test (Ebbeling et al., 1990), instead of the modified Bruce protocol, and thus were excluded from current analyses. Due to the differences in VO_2_ peak protocols, aerobic fitness between YA and OA was not compared statistically.

### Statistical Analyses

Data were screened for missing cells; 9% of the data were missing, and subsequently coded as missing values. Data were also screened for extreme outliers: values beyond <Q1–1.5*IQR or >Q3 + 1.5*IQR. Statistical analysis was completed using SPSS (IBM SPSS Statistics for Macintosh, version 24.0; IBM Corp., Armonk, NY, USA). For all statistical analyses, a *p* value (two-tailed) of <0.05 was considered significant, unless otherwise stated. Missing cells and outliers were excluded pairwise. Normality was assessed by histograms and significance on the Kolmogorov-Smirnov, and homogeneity of variance was assessed using Levene’s test.

A large portion of the data was not normally distributed (YA: 3/5; OA: 2/5) or did not have equal variance across groups (2/5). As such, non-parametric tests were used for all analyses. Two YA and five OA were identified as outliers in general recognition memory. One OA was identified as an outlier in VO_2_ peak performance and three OA were identified as outliers in high-interference memory. All outliers were excluded pairwise from subsequent analyses.

To assess differences in high-interference memory and general recognition memory between age groups (YA, OA), rank transformation of raw data was performed. Subsequently, group means were compared using a Mann-Whitney U.

Associations between memory performance and age were evaluated using Spearman’s correlations (two-tailed), with age as both a continuous and a grouping variable (YA, OA). Among OA, non-parametric partial correlations were used to evaluate the associations between memory performance and age, with MoCA score included as a covariate. The relationship between aerobic fitness and memory performance was assessed using non-parametric partial correlations, with age, sex, and BMI entered separately as covariates.

## Results

### Aging and Memory

Table 1 displays mean high-interference and general recognition memory scores for YA and OA. YA outperformed OA on high-interference memory (*U* = 1265.0, *p* < 0.001), but not general recognition memory (*U* = 2974.5, *p* = 0.45).

**Figure 2:**
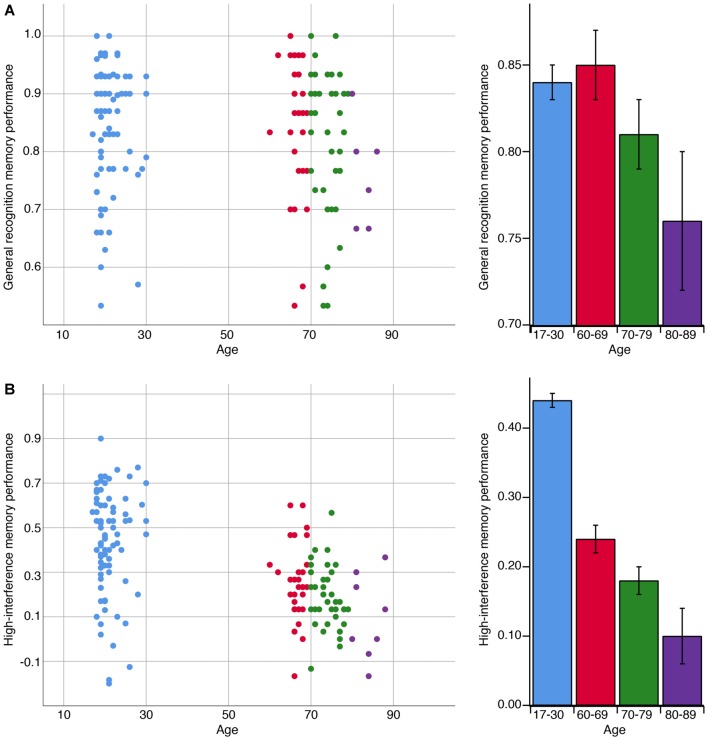
The association between age in years and memory performance on general recognition and high-interference memory tasks. **(A)** A negative correlation was observed with age and general recognition memory performance but only among adults over 60 years (*r*_s(75)_ = −0.27, *p* = 0.02). General recognition memory performance reflects the ability to correctly identify repetitions items as “Old” [p(“Old”|Repeat image) − p(“Old”|Foil image)]. **(B)** In contrast, a negative correlation was observed with age and high-interference memory performance across the lifespan (*r*_s(162)_ = −0.54, *p* < 0.001). High-interference memory performance reflects the ability to correctly identify lure items as “Similar” [p(“Similar”|Lure image) − p(“Similar”|Foil image)]. *Note*: bar graphs depict mean performance per age bin: 17–30 years old, *n* = 95; 60–69 years old, *n* = 33; 70–79 years old, *n* = 41; 80–89, *n* = 8. Error bars represent standard error of the mean.

The decline in memory across age is depicted in Figure 2. Across all age groups, we observed a negative correlation between age and high-interference memory (*r*_s(162)_ = −0.54, *p* < 0.001) and no correlation between age and general recognition memory (*r*_s(158)_ = −0.09, *p* = 0.25). For OA, we observed a negative correlation between the participants’ age and their performance on both high-interference memory (*r*_s(77)_ = −0.28, *p* = 0.01) and general recognition memory (*r*_s(75)_ = −0.27, *p* = 0.02). These relationships remained significant when controlling for MoCA scores (high-interference: *r*_s(76)_ = −0.29, *p* = 0.01; general recognition: *r*_s(74)_ = 0.26, *p* = 0.02). For YA, the participants’ age was not correlated with performance on either memory task (high-interference: *r*_s(83)_ = −0.05, *p* = 0.67; general recognition: *r*_s(81)_ = −0.09, *p* = 0.44).

### Aerobic Fitness and Memory

**Figure 3:**
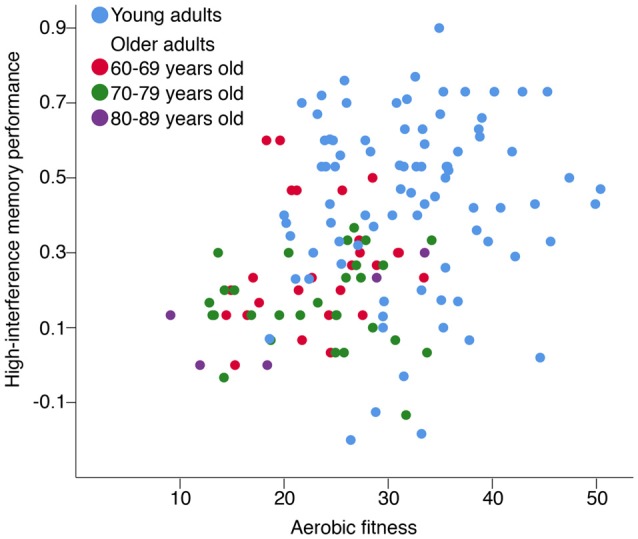
The association between high-interference memory and aerobic fitness. Aerobic fitness was positively correlated with high-interference memory performance for older adults (OA; *r*_s(58)_ = 0.27, *p* = 0.035), but not for young adults (YA; *r*_s(82)_ = 0.08, *p* = 0.45). *Note*: Aerobic fitness was assessed using different predictive test of peak oxygen uptake (VO_2_ peak; [mL/(kg•min)]) for YA and OA. High-interference memory performance reflects the ability to correctly identify lure items as “Similar” [p(“Similar”|Lure image) − p(“Similar”|Foil image)].

Aerobic fitness was positively correlated with better high-interference memory performance for OA (*r*_s(58)_ = 0.27, *p* = 0.035), but not for YA (*r*_s(82)_ = 0.08, *p* = 0.45; Figure 3). This relationship between aerobic fitness and high-interference memory for OA remained significant when controlling for age (*r*_s__(57)_ = 0.26, *p* = 0.047), sex (*r*_s(57)_ = 0.26, *p* = 0.049), and BMI (*r*_s(57)_ = 0.31, *p* = 0.018). In contrast, there were no correlations between aerobic fitness and general recognition memory for either age group (YA: *r*_s(80)_ = 0.17, *p* = 0.13; OA: *r*_s(57)_ = −0.15, *p* = 0.26).

## Discussion

The present study examined the association between aging and aerobic fitness on different aspects of memory. High-interference memory declined with advancing age. Aerobic fitness among the OA was associated with better high-interference memory performance suggesting the trajectory of decline in high-interference memory may be altered by aerobic exercise. In contrast, general recognition memory did not begin to decline until after 60 years of age and was not associated with aerobic fitness.

The results help to clarify a puzzle from prior work. Namely, in Stark et al. (2013), high-interference memory appeared plateau at age 60 in some individuals, despite the progressive atrophy of the hippocampus well beyond that age (Ziegler et al., 2012). Here, we did not observe evidence of a plateau. Instead, high-interference memory progressively declined from young adulthood and beyond 80 years of age (Figure 2). Although we used the same task and had a similar age range among the YA and OA, a key difference was that our sample was more than double in size than that of Stark et al. (2013). This afforded us with greater power to detect memory decline with advancing age.

We also observed age-related decline in general recognition memory among the OA, which is not typically reported (Mitchell and Bruss, 2003; Danckert and Craik, 2013; Holden et al., 2013; Stark et al., 2013). However, in contrast to high-interference memory, these age differences did not emerge until later in life. This likely reflects the tendency for OA to rely more on gist and familiarity-based memory, at the expense of encoding highly specific details needed for high-interference memory (Ferguson et al., 1992; Koutstaal et al., 1999). This bias in processing may expose deficits in high-interference memory earlier. Furthermore, the more distributed network that general recognition memory recruits may facilitate compensatory processing if one part of the network becomes dysfunctional (Yonelinas et al., 2005; Neufang et al., 2006). That said, eventually the accumulated damage with aging would be so great that it would impair performance, as seen in the oldest old of our sample.

In addition to the effects of age on memory, aerobic fitness was associated with high-interference memory in OA. This is consistent with prior research in animal models, which has shown that exercise can partially reverse the age-related decline in neurogenesis (van Praag et al., 2005), which may help to preserve high-interference memory. Moreover, exercise has been shown to improve high-interference memory in YA (Déry et al., 2013; Heisz et al., 2017; Suwabe et al., 2017b), suggesting that the impact of aerobic exercise on the brain may be preserved across species and the human lifespan. Furthermore, the lack of association between aerobic fitness and general recognition memory suggests that aerobic fitness may selectively benefit high-interference memory. Indeed, aerobic exercise induces structural changes in the hippocampus, which includes the up-regulation of neurogenesis (van Praag et al., 1999a,b; Olson et al., 2006; Fabel et al., 2009; So et al., 2017) and increases in cerebral blood volume (Pereira et al., 2007). Because high-interference memory recruits the hippocampus to reduce interference among highly similar stimuli, the impact of exercise-induced structural changes to the hippocampus may be more apparent for this aspect of memory.

The lack of association between aerobic fitness and high-interference memory in YA is likely related to the low activity levels of our sample. The archival datasets were taken from baseline testing sessions and done prior to an exercise intervention. An inclusion criteria for the intervention was that all participants engage in less than 1 h of vigorous physical activity per week. Critically, individuals who engaged in low to moderate intensity exercise were still eligible to participate. Although this may have been a sufficient stimulus for OA to show a positive association with memory, it may not have been enough to evoke a change in YA. OA primarily engage in physical activity at a low intensity (e.g., walking; Rafferty et al., 2002), which reduces their rate of hippocampal atrophy (Varma et al., 2015) and risk of dementia (Fenesi et al., 2017). In contrast, prior work in YA has used high-intensity interval exercise to demonstrate an effect on high-interference memory (Déry et al., 2013; Heisz et al., 2017). Given that YA are already functioning at a higher level and have yet to experience atrophy of the hippocampus, they may require a greater intensity of exercise to alter memory. More research is needed to examine the potential for an age-specific dose-response relationship between aerobic fitness and memory.

While this study makes important contributions to our understanding of aging effects on memory, it is not without limitations. Separate protocols were used to assess aerobic fitness in YA and OA, and therefore, we could not directly compare aerobic fitness between groups. Moreover, our exclusion criteria for sedentary participants focused on vigorous activity. Low and moderate intensity exercises were not considered and may be responsible for the age-specific effect of fitness on memory function. A broader spectrum of aerobic fitness, including both sedentary and physically active participants, would provide further insight into the relationship between memory and aerobic fitness. Additionally, our study did not take into account target-lure similarity, the degree of mnemonic similarity between the target and the lure in the MST (Suwabe et al., 2017a). Previous research suggests that the relationship between aerobic fitness and performance on the MST in YA is influenced by the degree of target-lure similarity (Suwabe et al., 2017a). Thus, future studies should include target-lure similarity to elucidate this relationship.

In conclusion, we observed differential effects of aging on high-interference and general recognition memory performance. High-interference memory progressively declined with age and there was no indication of a plateau in performance among the OA. OA with higher aerobic fitness had better high-interference memory, suggesting this may be a key variable influencing the rate of memory decline. In contrast, general recognition memory did not begin to decline until after the age of 65, and performance was not associated with aerobic fitness. This may be because deficits in general recognition memory result from wider-spread damage to the brain that cannot be recovered solely by lifestyle factors. More research is needed to understand the interactive effects of aging and lifestyle on memory.

## Author Contributions

AMB, ALM and JJH: data analysis, wrote the article, manuscript revisions. AK: data collection and manuscript revisions.

## Conflict of Interest Statement

The authors declare that the research was conducted in the absence of any commercial or financial relationships that could be construed as a potential conflict of interest. The reviewer SAJ and the handling editor declared their shared affiliation.
